# Frequency-modulated continuous waves controlled by space-time-coding metasurface with nonlinearly periodic phases

**DOI:** 10.1038/s41377-022-00973-8

**Published:** 2022-09-14

**Authors:** Jun Chen Ke, Jun Yan Dai, Jun Wei Zhang, Zhanye Chen, Ming Zheng Chen, Yunfeng Lu, Lei Zhang, Li Wang, Qun Yan Zhou, Long Li, Jin Shan Ding, Qiang Cheng, Tie Jun Cui

**Affiliations:** 1 Institute of Electromagnetic Space, Southeast University, 210096 Nanjing, China; 2 State Key Laboratory of Millimeter Waves, Southeast University, 210096 Nanjing, China; 3 School of Electronic Engineering, Xidian University, 710071 Xi’an, China

**Keywords:** Metamaterials, Microresonators, Microwave photonics, High-harmonic generation

## Abstract

The rapid development of space-time-coding metasurfaces (STCMs) offers a new avenue to manipulate spatial electromagnetic beams, waveforms, and frequency spectra simultaneously with high efficiency. To date, most studies are primarily focused on harmonic generations and independent controls of finite-order harmonics and their spatial waves, but the manipulations of continuously temporal waveforms that include much rich frequency spectral components are still limited in both theory and experiment based on STCM. Here, we propose a theoretical framework and method to generate frequency-modulated continuous waves (FMCWs) and control their spatial propagation behaviors simultaneously via a novel STCM with nonlinearly periodic phases. Since the carrier frequency of FMCW changes with time rapidly, we can produce customized time-varying reflection phases at will by the required FMCW under the illumination of a monochromatic wave. More importantly, the propagation directions of the time-varying beams can be controlled by encoding the metasurface with different initial phase gradients. A programmable STCM prototype with a full-phase range is designed and fabricated to realize reprogrammable FMCW functions, and experimental results show good agreement with the theoretical analyses.

## Abstract (web summary)

A novel theoretical framework and method, which is based on a novel space-time-coding metasurface (STCM) with nonlinearly periodic phases, is proposed to generate frequency-modulated continuous waves (FMCWs) and dynamically control their spatial propagation behaviors simultaneously.

## Graphical abstract

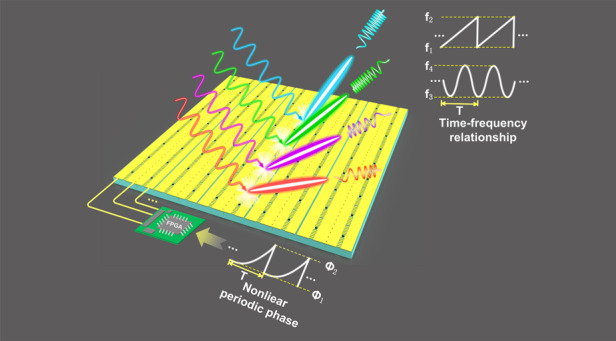

## Introduction

Over the past decades, metamaterials and metasurfaces have demonstrated powerful abilities to manipulate the properties of electromagnetic (EM) waves and wavefronts^1–4^. As a kind of two-dimensional (2D) patterned interface, metasurfaces can be arbitrarily controlled to achieve exotic EM phenomena that are not possible in nature, as particularly valued by the applications such as beamforming, polarization conversion, and holographic imaging^5–8^. The local control of the reflection/transmission features at different positions of the metasurfaces is accomplished by altering the shapes, dimensions, and spatial alignments of meta-atoms, which offers unprecedented degrees of freedom to synthesize the amplitude and phase profiles. However, the metasurfaces have only fixed functions once they are fabricated. To tackle this problem, the concept of digital coding and programmable metasurfaces was proposed^9^, and the corresponding radiation/scattering characteristics of the metasurfaces are highly related to the coding sequences of finite types of meta-atoms. Once tunable devices are incorporated in the meta-atoms, such as positive-intrinsic-negative (PIN) diode, varactor, graphene, and liquid crystals, it is feasible to develop reprogrammable platforms using a single metasurface for completely different functions switched in real-time, such as dynamic beam generations, beam scanning, and scattering reductions^10–14^.

Although the metasurfaces have aroused great interest in both scientific and engineering communities, they are still limited by the constraints of Lorentz reciprocity in manipulating the EM waves. To overcome this difficulty, time-modulated metasurfaces have been further developed, with the constitutive parameters varying in the time and space domains to break the Lorentz reciprocity, thus bringing a new degree of freedom for manipulating the frequency spectra of the EM waves^15–18^. Plenty of novel physical phenomena and applications have been inspired, including non-reciprocal antennas, Doppler cloaks, frequency conversion, and compression of lines of force^14,19–22^. In addition, as an alternative, space-time modulation can be implemented by modulating the parameters of different circuit components, so that the space-time modulation was first introduced into the circuit system. In this way, researchers have successively achieved excellent circuit characteristics such as accumulation of EM energy, wireless transfer enhancement of power and information, and power combiner of EM waves^23–25^. However, the proposed space-time-modulated metasurfaces are mostly based on theoretical analyses or numerical simulations, and there are considerable difficulties and limitations in experimental realizations.

Recently, nonlinear frequency modulations based on time-domain-coding metasurfaces and space-time-coding metasurfaces (STCMs) have attracted considerable attention. Different from the previous *analog* time-domain modulations of EM/circuit characteristic parameters, by elaborately designing the reflection amplitudes/phases in different time slices in a digital-coding way, it is possible to tailor the propagation behaviors of nonlinear harmonics^18,26–29^. Some unique applications based on STCMs have been reported, including independent control of multiple harmonics, high-efficiency frequency synthesizer, space-time modulation, and nonlinear polarization synthesis^30–37^. Direct digital information modulations can also be realized by STCM, which enables us to construct new architecture wireless communication transmitters^26,38–42^. It can be seen that the metasurface has gradually evolved from a tool for wave manipulations to an integrated information system. As a vital part of the system, the generation of transmitting temporal signals based on STCMs is still unconsidered.

Furthermore, in engineering applications, especially in the field of modern radar systems, an agile synthesis scheme for continuous-time waveforms is a longing for applications. With the increasing complication and diversification of targets under detection, modern radar technology should be developed towards higher speeding and ranging accuracies. Hence, the pulse compression technique is especially favored with large time-width and large bandwidth to solve the conflict between the time-width and bandwidth^43–48^. The typical pulse compression signals are intra-pulse frequency-modulated continuous wave (FMCW) signals, whose instantaneous frequencies change with time. Currently, the FMCW signal generation primarily relies on voltage-controlled oscillators (VCOs) or direct digital synthesis (DDS) technologies^49–52^. However, both solutions are operating at the circuit level, which requires high-performance devices to achieve excellent signal qualities. Meanwhile, advanced digital signal processors (DSPs) are accompanied by the modulation/demodulation processes. To reduce the device cost and facilitate system integration, some researchers pointed out that analog signal processing can be implemented directly with the metasurface phasors^53–55^, which is much advantageous for handling broadband information at high frequencies. Such architectures are very simple and have low costs compared to the traditional DSP devices, and provide a competitive plan for constructing new electronic systems. However, the frequency synthesizers and antennas cannot be effectively integrated together, which greatly hinders the development of such new-concept systems.

To this aim, here we propose a theoretical framework and methodology to realize typical FMCW signals and manipulate their propagation directions in spatial domain simultaneously via the STCM. A novel STCM with full-phase-range modulation is presented and fabricated to validate the proposed theory and methodology. The measurement results are in good agreement with the theoretical analyses, showing good application potentials of the proposed scheme in future radar systems.

## Results

### Generation of FMCWs based on STCM

**Fig. 1:**
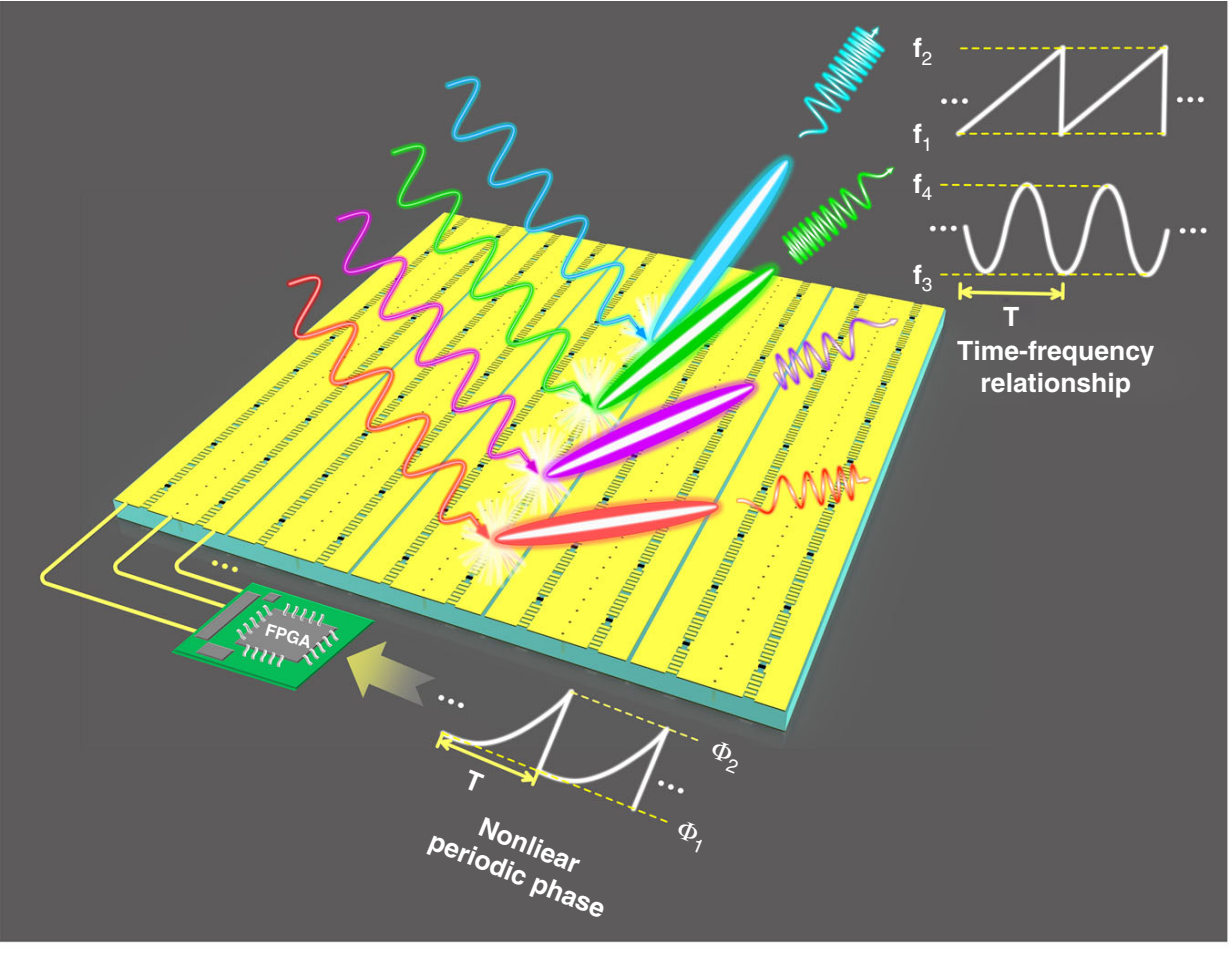
Concept illustration of waveform generations and beam shaping of various FMCWs by a programmable STCM with nonlinearly periodic phases. Under the control of a FPGA that offers external nonlinear periodic voltage control signals, the different types of FMCW signals can be synthesized as required at the same STCM platform. By further optimizing the initial voltage distributions among different regions of the metasurface, the propagation directions of the FMCW beams can be manipulated freely.

The frequency-modulated (FM) signal is usually obtained by linear or nonlinear frequency modulation of a single-tone signal, whose instantaneous frequency *f* is varied with time. Thus the phase of the FM signal can be expressed by the calculus of the instantaneous frequency function $f\left( t \right)$^6^:

1 $$S\left( t \right) = A( t )\exp [ {j\varphi ( t )} ] = A( t )\exp [ {j \cdot 2\pi {\smallint} {f( t )dt} } ]$$

where $A\left( t \right)$ and $\varphi \left( t \right)$ are the amplitude and phase of the transmitting waveform $S\left( t \right)$, respectively. From Eq. (1), it is easy to find that we can synthesize the temporal transmitting waveform of FMCW by changing the instantaneous reflection phase of STCM dynamically. We present the concept illustration of the FMCW waveform generation in Fig. 1. Under the control of a field programmable gate array (FPGA) that offers external time-varying biasing voltages of meta-atoms, we can obtain a dynamic reflection coefficient with the STCM: $\Gamma \left( t \right) = \left| {\Gamma (t)} \right|{\mathrm{exp}}\left[ {j\varphi \left( t \right)} \right]$, in which $\left| {\Gamma (t)} \right|$ and $\varphi \left( t \right)$ are the amplitude and phase of $\Gamma \left( t \right)$, respectively. If we get accurate phase responses according to the demands of linear or nonlinear frequency modulation functions, it is possible to synthesize different types of FMCWs as required at the same platform.

Specifically, when the metasurface is excited by a monochromatic wave at the normal incidence with the carrier frequency of $f_c$ and the electric field $E_i\left( t \right) = {\mathrm{exp}}(j2\pi f_ct)$, the echo wave $E_r(t)$ can be represented as:

2 $$E_r\left( t \right) = E_i\left( t \right) \cdot \Gamma \left( t \right) = \left| {\Gamma (t)} \right|{\mathrm{exp}}\left\{ {j\left[ {2\pi f_ct + \varphi \left( t \right)} \right]} \right\}$$

Assume that we have a highly reflective metasurface with the reflection amplitude $\left| {\Gamma (t)} \right|$ equal to 1, $\varphi \left( t \right)$ is a periodic function of time as follows

3a $$\varphi \left( t \right) = \mathop {\sum }\limits_{n = - \infty }^{ + \infty } \varphi _0\left( {t - nT} \right)$$

where

3b $$\varphi _0\left( t \right) = \varphi \left( t \right)\left[ {\varepsilon \left( t \right) - \varepsilon \left( {t - T} \right)} \right]$$

Note that $\varphi _0\left( t \right)$ is a portion of $\varphi \left( t \right)$ in a period *T*, and $\varepsilon \left( \cdot \right)$ is the step function. When the phase difference of $\varphi _0\left( t \right)$ at the starting and ending time of each period is the integral multiple of $2\pi$

4 $$\Delta \varphi = \varphi _0\left( T \right) - \varphi _0\left( 0 \right) = 2m\pi$$

in which *m* is an integer, the phase continuity can be ensured during the modulation in Eq. (2), indicating that the reflected wave is a phase-continuous wave without sharp phase jumps. In addition, if $\varphi _0\left( t \right)$ is differentiable in the range of [*0*, *T*], we can calculate the instantaneous frequency $f(t)$ of the reflected wave as:

5 $$f(t) = f_c + \frac{1}{{2\pi }}\mathop {\sum }\limits_{n = - \infty }^{ + \infty } \frac{{d[\varphi _0\left( {t - nT} \right)]}}{{dt}}$$

From Eq. (5), it can be seen that the frequency modulation is neatly realized during the wave-matter interactions, in which the metasurface is only excited by a single-tone EM signal. Both linear and nonlinear frequency modulations can be implemented on the same platform, and the modulation functions can be programmed by software^31,36–49^. We remark that we do not need the frequency synthesizing module used in the traditional superheterodyne systems, and hence greatly reduce the system costs and system complexity. During the generation of FMCW waveform based on STCM, it is worth noting that Eq. (4) should be satisfied, and the reflection phase $\varphi \left( t \right)$ should be differentiable in each period as well.

### Linear and nonlinear FMCW signal generations based on STCM

For the linear frequency modulation, where $f(t)$ is a linear function of time, the reflection phase $\varphi _0\left( t \right)$ should be a quadratic function from Eq. (1). From Eq. (4), we can get the general expression of $\varphi _0\left( t \right)$ of the linear FMCW as:

6 $$\varphi _0\left( t \right) = {{\Phi }}_0 + \left( {\frac{{2m\pi }}{T} - p\pi T} \right)t + p\pi t^2,0 \le t \le T$$

in which ${{\Phi }}_0$ is the initial phase, *p* is the linear FM slope, and *T* is the period of the waveform. For simplicity, we assign ${{\Phi }}_0 = 0$ in all linear and nonlinear frequency modulations.

**Fig. 2:**
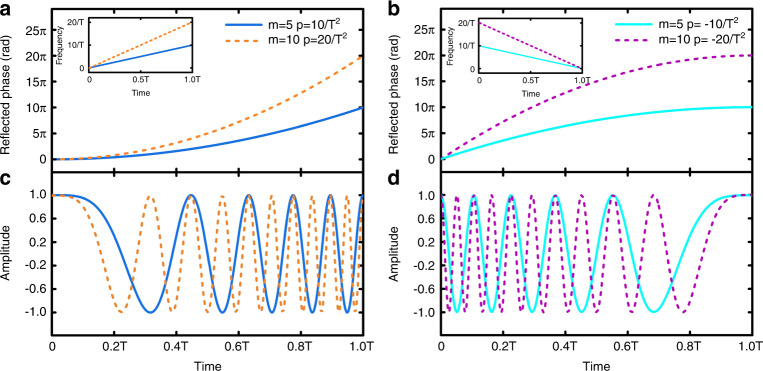
Generations of linear FMCW signals based on STCM with nonlinear periodic phases. **a**, **b** Four types of reflection phase responses $\varphi _0\left( t \right)$ under different combinations of the frequency slope *p* and the repetition number *m*, in which the insets show the corresponding time-frequency diagrams after down-conversions. **c**, **d** The linear FMCW waveforms of the four cases in baseband

The instantaneous frequency of the waveform is $f_0\left( t \right) = f_c + \left( {\frac{m}{T} - \frac{{pT}}{2}} \right) + pt$ by taking the derivative of $\varphi _0\left( t \right)$. On one hand, the item $\left( {\frac{m}{T} - \frac{{pT}}{2}} \right)$ offers an additional frequency offset to the carrier frequency of the incident wave $f_c$, so that we can control the initial frequency of the FMCW signal freely. On the other hand, this item ensures the phase continuity condition as demanded by Eq. (4), which is helpful to smooth the frequency response curve. The reflected phase of STCM needs to traverse $2m\pi$ in one period, indicating that the FMCW waveform goes through *m* times of oscillations. To intuitively display the properties of the STCM-based linear FMCW signal, Fig. 2a and b illustrate four types of $\varphi _0\left( t \right)$ under different combinations of *m* and *p: m* = 5 and *p* = $\frac{{10}}{{T^2}}$, *m* = 5 and *p* = $- \frac{{10}}{{T^2}}$, *m* = 10 and *p* = $\frac{{20}}{{T^2}}$, as well as *m* = 10 and *p* = $- \frac{{20}}{{T^2}}$, respectively. The insets show the corresponding time-frequency diagrams, which have been down-converted from the incident frequency $f_c$ to the baseband. Figure 2c and d exhibit the corresponding linear FMCW waveforms in one period, in which the displayed waveforms are demodulated from $f_c$ to the basebands. Owing to the highly reflective feature, the reflected signal can be regarded as a constant envelope signal. By changing the frequency slope *p* and the repetition number *m* of STCM, the initial frequency and the waveform of the linear FMCW can be effectively controlled.

**Fig. 3:**
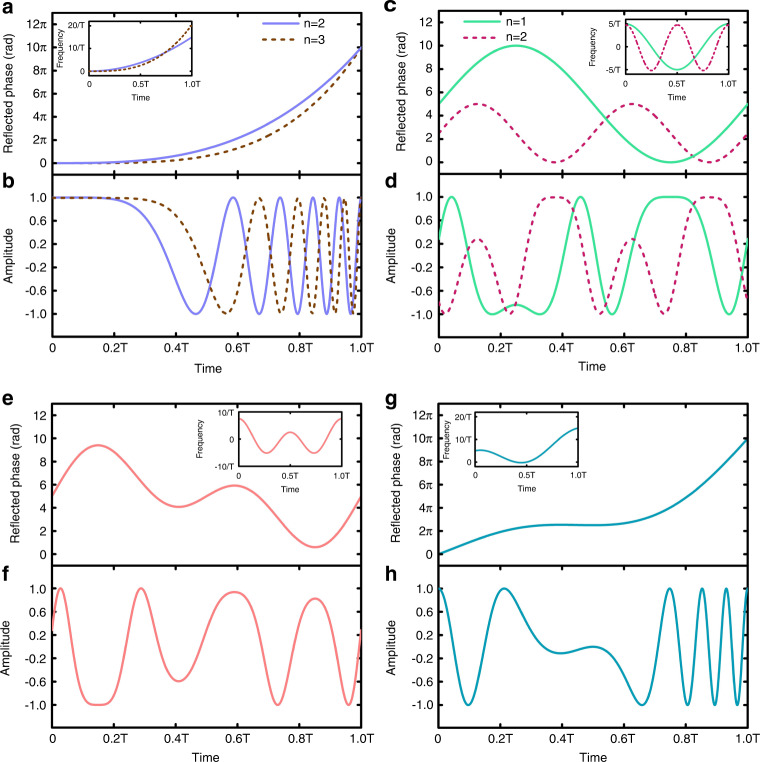
Generations of nonlinear FMCW signals based on STCM with nonlinear periodic phases. **a**, **c**, **e**, **g,** Reflection phase responses $\varphi _0\left( t \right)$ for Polynomial FMCWs under two different power index *n* (**a**), Sinusoidal FMCWs under two different coefficient *n* (**c**), Double sinusoidal FMCW (**e**), and S-shaped FMCW (**g**), in which the insets of **a**, **c**, **e**, **g** show the corresponding time-frequency diagrams after down-conversions. **b**, **d**, **f**, **h** The nonlinear FMCW waveforms of the four types in baseband.

In contrast to the linear FMCW, nonlinear frequency modulations are also frequently encountered for radar applications^56–58^, hence we need to investigate the nonlinear FMCW generations. Three typical nonlinear FMCW signals are considered: polynomial, sinusoidal, and S-shaped FMCWs. Detailed analytical expressions of the waveforms can be found in Materials and methods. Figure 3a and b display the required phases $\varphi _0\left( t \right)$ of STCM and the corresponding baseband waveforms for the quadratic (*n* = 2, *m* *=* 5, and *p* = $\frac{{15}}{{T^3}}$) and cubic (*n* = 3, *m* *=* 5, and *p* = $\frac{{20}}{{T^4}}$) polynomial FMCWs, respectively, and the insets demonstrate the corresponding time-frequency diagrams. Figure 3c and d give the same curves for sinusoidal FMCWs with (*n* = 1, *m* = 0, $\Delta f = \frac{5}{T}$) and (*n* = 2, *m* = 0, $\Delta f = \frac{5}{T}$), respectively. The cases of double sinusoidal FMCW (*k* = 2, *n* = 1, *m* = 0, and $\Delta f_2 = 2\Delta f_1 = \frac{5}{T}$) and the S-shaped FMCW (*n* = 1, *m* = 5, *p* = $\frac{{10}}{{T^2}}$ and $\Delta f = \frac{5}{T}$) are illustrated in Fig. 3e–h, respectively.

### Dynamic beam shaping of the STCM-based FMCWs

Besides generating the FMCW signals, STCM can engineer the beam propagation behaviors of the transmitted FMCW signals in the space domain at the same time. Such characteristics are especially favored for system integration since the metasurface serves as a combination of phased array antenna and frequency synthesizer. To realize the far-field beamforming with the metasurface, it is necessary to construct specific amplitude and phase profiles for the composing meta-atoms according to the antenna theory^59^. Since the reflection loss at the interface of the meta-atom is not very large, we only need to optimize the phase distributions of the whole metasurface to control the beam deflections. Suppose that STCM is made of *N* columns with a column width of *d*. Each column has an independent initial phase ${{\Phi }}_{0,n}$, where the subscript *n* represents the column number. Herein, we introduce an initial phase gradient along the metasurface, thus ${{\Phi }}_{0,n}$ can be expressed as ${{\Phi }}_{0,n} = {{\Phi }}_{0,1} + (n - 1)\varphi _{{\mathrm{adj.}}}$, where $\varphi _{{\mathrm{adj.}}}$ is the phase difference between the adjacent two columns. Thereupon, the initial reflected electric field of the *n-*th column could be written as $E_{r,n}(t) = E_{r,1}(t){\mathrm{exp}}\{ {j[ {( {n - 1} )\varphi _{{\mathrm{adj.}}}} ]} \}$. In the case of the FM deviation $\Delta f \ll f_c$, imitating the analysis method of the uniform linear antenna-arrays^60^, we can calculate the beam deflection angle $\theta _c$ of FMCW as:

7 $$\theta _c = {\mathrm{arcsin}}\left( {\frac{c}{{2\pi f_cd}}\varphi _{{\mathrm{adj.}}}} \right)$$

in which *c* is the light speed in vacuum. By dynamically adjusting the phase difference $\varphi _{{\mathrm{adj.}}}$, the reflected phase gradient will be continuously changed, and the beam deflection angle can be varied in time.

It is worth noting that, here, we only change the initial phases of the meta-atoms to achieve the desired phase patterns, which implies that the initial phase ${{\Phi }}_0$ varies at different positions of the metasurface. But it has no impact on the system performance because, in the FMCW system, we detect the targets based on the frequency difference between the transmitted and received signals, which is not sensitive to the initial phase ${{\Phi }}_0$ of FMCW in Eq. (6)^6^.

### Design of the STCM structure

**Fig. 4:**
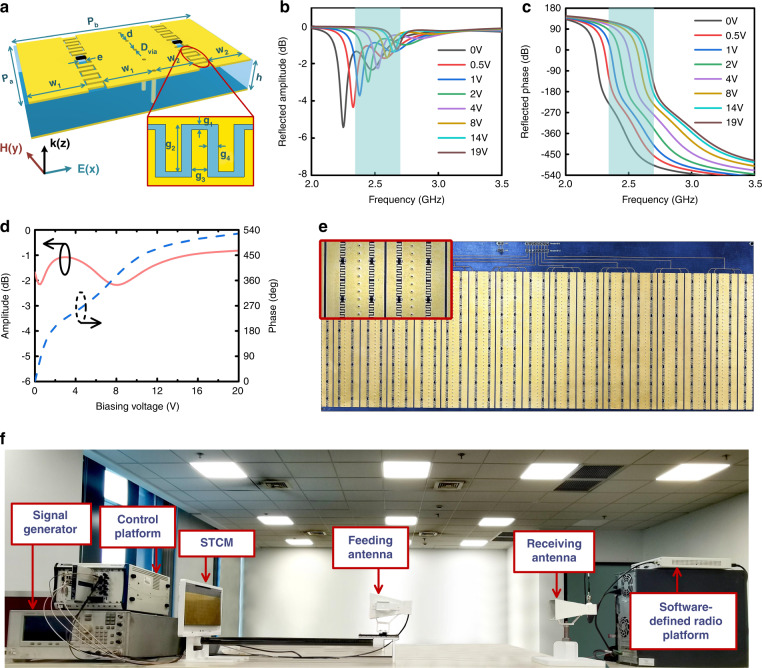
Design details, EM properties, and measurement setup of the STCM. **a** Design of meta-atoms for STCM, where the inset shows the details of the interdigital structures. **b**, **c** The simulated refection amplitude and phase spectra under different biasing voltages, in which the highlighted region stands for the bandwidth with over 360^o^ phase coverage. **d** The measured amplitude and phase responses under different biasing voltages from 0 to 19 V at 2.6 GHz. **e** Photograph of the fabricated metasurface prototype, where the inset shows the details of the meta-atoms. **f** Experimental setup configuration

To validate the theoretical analysis, we design a reflective STCM with a full-phase range. Each element of STCM consists of a meta-atom and its mirror structure along *x* direction. As illustrated in Fig. 4a, the meta-atom includes a dielectric substrate (*F4B*, ${\it{\epsilon }}_r = 2.2$, and ${\mathrm{tan}}\;\delta = 0.0015$), a metallic ground, and an upper patch with complementary interdigital structures. The varactor diodes (SMV-1405, Skyworks, Inc.) are incorporated into the meta-atoms to provide reconfigurability of the meta-atoms. The equivalent circuit model and detailed effective circuit parameters can be found in ref. ^40^. Compared to previously reported full-phase-coverage meta-atoms^42,61^, the proposed one employs the interdigital structures as the distributed capacitors to replace the chip capacitors, which can reduce the risk of device inconsistency and improve the stability of the EM responses. Several metallic via-holes are used in the middle region to provide reverse DC biasing voltages to the varactors. The resonant frequency of the meta-atom can be gradually tuned by increasing the biasing voltage^62^, thus achieving a large phase tuning range over 360°. With the help of EM optimizations, the dimensions of the meta-atom in Fig. 4a are finally determined as: $P_a$ = 15 mm, $P_b$ = 23.8 mm, $w_1$ = 4 mm, $w_2$ = 5.5 mm, $D_{{\mathrm{via}}}$ = 0.5 mm, *d* = 3 mm, *e* = 1 mm, *h* = 4 mm, $g_1$ = 0.2 mm, $g_2$ = 1.8 mm, $g_3$ = 0.6 mm, and $g_4$ = 0.4 mm.

To obtain the reflected amplitude and phase responses of the meta-atom, full-wave EM simulations are performed by using a commercial EM solver (CST Microwave Studio 2019). The boundary conditions of the meta-atom along the *x* and *y* directions in Fig. 4a are set as perfectly electric conductor (PEC) and perfectly magnetic conductor (PMC), respectively, to mimic a two-dimensional infinite meta-atom array. An *x*-polarized EM wave is normally incident upon the meta-atom as the excitation. During the simulation, the varactor diodes are described as an equivalent circuit model^40^. The simulated reflection amplitude and phase spectra are illustrated in Fig. 4b and c. When the biasing voltage increases from 0 to 19 V, the resonant frequency tends to shift up as expected, leading to a large phase range exceeding 360° from 2.35 to 2.7 GHz. Finally, we choose 2.6 GHz as the working frequency due to the balance between the phase range and reflection loss.

### Experimental results

To evaluate the performance of the proposed STCM, a sample with 8 × 8 elements was designed and manufactured using the standard printed circuit board (PCB) technology, as shown in Fig. 4e. The overall size of the sample is 385.35 × 153.25 mm. To simplify the feeding circuit of the metasurface, the diodes in the same column share identical biasing voltage, and thus the working states of these diodes can be controlled synchronously. The measured reflection coefficients under various bias voltages at 2.6 GHz are presented in Fig. 4d. We note that, as the biasing voltage grows gradually from 0 to 19 V, the reflection phase of the metasurface is continuously controlled in a range of nearly 520°, which is sufficient to meet the requirement of the spatial signal generation. The measured reflection loss is slightly larger than the simulated result but remains smaller than 2.5 dB as the biasing voltage changes. The fluctuation of the reflection amplitude is below 1.3 dB. The deviation between the simulated and measured results is primarily ascribed to the processing tolerance, deviations of material and diode parameters, and the finite size of the metasurface. Nevertheless, the manufactured STCM can be employed in subsequent experiments for generating the FMCW signals and manipulating the EM waves.

Firstly, we make experiments to generate several different types of FMCW signals using the fabricated STCM sample. The experimental configuration is illustrated in Fig. 4f. During experiments, the incident single-tone signal is generated by a microwave signal generator Agilent E8257D and radiated by a horn antenna to illuminate the metasurface at the normal direction. A control platform is used to provide arbitrarily periodic control signals, and these control signals can be converted to voltage waveforms to drive the metasurface for generating the required instantaneous phase curves. Through the procedure, STCM can reradiate the FMCW signals to free space. In the receiving part, a horn antenna is used for receiving the FMCW signals and transmitting them to a software-defined radio reconfigurable device (NI USRP-2943R, National Instruments Corp.), in which the FMCWs are down-converted to the baseband waveforms. Then the baseband waveform data are conveyed to a computer for postprocessing. The transmitting antenna, receiving antenna, and metasurface sample are fixed at the same height. All instruments are synchronized by phase stable cables to obtain stable waveform data. Based on the relationship between the voltage and phase in Fig. 4d, we can easily calculate the driving voltages of varactors to generate the periodic voltage waveforms. During the experiments, we adopt a high-resolution digital-to-analog converter (DAC) module and optimize the feeding circuit and meta-atom design to maintain the control signal integrity. We remark that if the demanded $\varphi _0\left( t \right)$ is out of the range from 0 to $2\pi$, an extra integer multiple of $2\pi$ should be added or subtracted to keep $\varphi _0\left( t \right)$ staying in that interval.

**Fig. 5:**
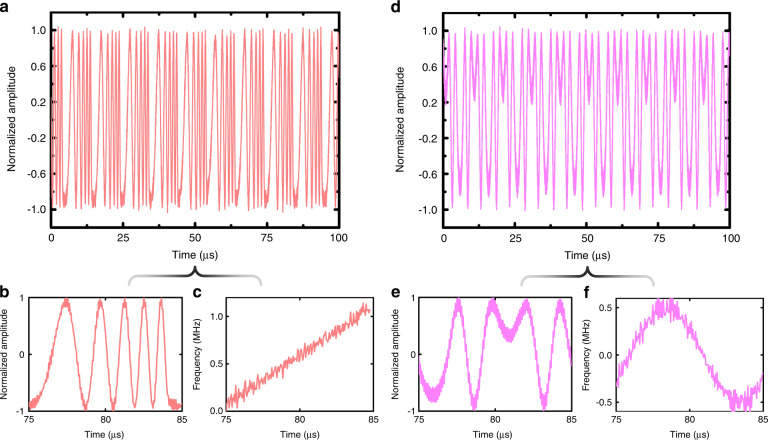
Measured results of the linear and sinusoidal FMCWs. **a**–**c** The measured baseband FMCW waveforms in ten periods (**a**) and one period (**b**), and the corresponding baseband time-frequency curve (**c**) for the linear FMCW. **d**–**f** The measured baseband FMCW waveforms in ten periods (**d**) and one period (**e**), and the corresponding baseband time-frequency curve (**f**) for the sinusoidal FMCW

In this experiment, two kinds of FMCWs—linear and sinusoidal—are generated with the fabricated sample. The period of the FMCW is set to 10 $\mu s$. From Eq. (6) and (9) in Materials and methods, we choose $\varphi _0\left( t \right) = 10\pi \left( {\frac{t}{T}} \right)^2$ and $\varphi _0\left( t \right) = 5\left[ {\sin \left( {2\pi \frac{t}{T}} \right) + 1} \right]$ for the linear and sinusoidal FMCWs, respectively. The measured baseband waveforms in the two cases are illustrated in Fig. 5a and d, respectively, which display the normalized waveforms in ten periods. For better observation, we extract the waveforms in one period in Fig. 5b and e, and give the corresponding time-frequency curves (see Fig. 5c and f) by taking derivatives of the measured $\varphi _0\left( t \right)$ with respect to time. We clearly see that the generated FMCWs are consistent with the theoretical ones with high accuracy. There are small signal burrs as found in the time-frequency curves. They are probably attributed to the distortion of control waveforms, the environmental EM interference, and the sampling rate limitation of the receiver.

**Fig. 6:**
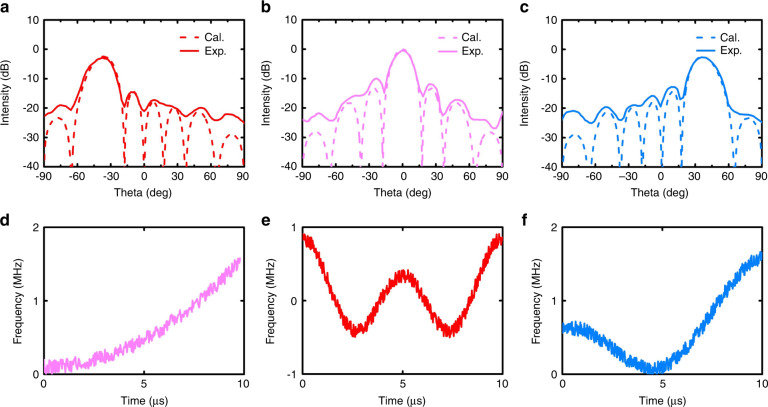
Measured results of the polynomial, double sinusoidal, and S-shaped FMCWs. **a**–**c** The calculated and measured scattering patterns in the space domain for the cases of polynomial, double sinusoidal, and S-shaped FMCWs. **d**–**f** The corresponding time-frequency curves for the waveforms in **a**–**c**

Finally, we experimentally demonstrate the capability of dynamic beam shaping for the FMCW signals using the STCM sample. The experimental configuration is the same as that in Fig. 4f, except that the measurement is carried out in the microwave chamber. The period of $\varphi \left( t \right)$ is also set as 10 *μs*. Since all eight columns of STCM are independently controlled, here we consider three FMCWs to deflect towards different angles in the horizontal direction: the polynomial FMCW with instantaneous phase $\varphi _0\left( t \right) = 10\pi \left( {\frac{t}{T}} \right)^3$, double sinusoidal FMCW with $\varphi _0\left( t \right) = \frac{5}{2}\left[ {\sin \left( {2\pi \frac{t}{T}} \right) + \sin \left( {4\pi \frac{t}{T}} \right) + 2} \right]$, and the S-shaped FMCW with $\varphi _0\left( t \right) = 10\pi \left( {\frac{t}{T}} \right)^2 + 5\sin \left( {2\pi \frac{t}{T}} \right)$. To realize the beam deflections, we adopt a 2-bit initial phase coding strategy and set four digits 0, 1, 2, 3 to represent the four initial phase values 0, $\frac{\pi }{2}$, *π*, $\frac{{3\pi }}{2}$, respectively. Subsequently, we preset the initial phase gradients for different beam deflections as follows: (3,2,1,0,3,2,1,0), (0,0,0,0,0,0,0,0), and (0,1,2,3,0,1,2,3). According to Eq. (7), the corresponding deflection angles for the abovementioned three FMCW signals are −37.3°, 0°, and 37.3°, respectively. Both theoretically predicted scattering patterns and measured scattering patterns at 2.6 GHz are presented in Fig. 6a–c for comparison. It can be observed that there are small errors in the sidelobes, as can be attributed to the finite dynamic range of the receiver, and the phase errors of the meta-atom due to the slight controlling waveform distortion. Nevertheless, their general tendencies and intensity distributions are consistent, which proves the feasibility of dynamic beam shaping for the FMCW signals. The corresponding waveforms are plotted in Fig. 6d–f, respectively. We can see that the experimental results agree well with the calculation ones, validating the powerful capabilities of the proposed STCM for simultaneous FMCW waveform generations and beam shaping.

## Discussions

In this work, we propose a novel approach to produce various FMCW signals using the STCM under the excitation of a monochromatic wave and realize dynamic beam shaping of the FMCW signals in free space. The time-varying phase responses of STCM are employed to synthesize the frequency modulation waveforms, which can remove the complex frequency synthesizers in traditional RF systems. Furthermore, the proposed framework can be employed in the millimeter-wave and multi-polarized regions by combining the advanced antenna-array optimization methods^63^. The proposed methodology has the advantages of simple hardware architecture, easy integration, and low cost. These important properties make it promising to find applications in microwave and optical detections and measurements.

## Materials and methods

### Polynomial FMCW

When the time-frequency relationship of FMCW is a polynomial function, it is defined as the polynomial FMCW. Here we consider a kind of polynomial FMCW, whose time-frequency curve is a power function with the power index of *n*. According to Eq. (4), the condition $\Delta \varphi = \varphi _0\left( T \right) - \varphi _0\left( 0 \right) = 2m\pi$ should be satisfied. Hence the time-varying reflected phase $\varphi _0\left( t \right)$ of the STCM is designed as:

8 $$\varphi _0\left( t \right) = \left( {\frac{{2m\pi }}{T} - \frac{{2p\pi }}{{n + 1}}T^n} \right)t + \frac{{2p\pi }}{{n + 1}}t^{n + 1},0 \le t \le T$$

in which *T* is the modulation period, and *n* and *p* are integers. The instantaneous frequency is $f_0( t ) = f_c + ( {\frac{m}{T} - \frac{p}{{n + 1}}T^n} ) + pt^n$. We remark that the linear FMCW is a special case with *n* = 1. Figure 3a and b demonstrate the required phases $\varphi _0\left( t \right)$ and the baseband waveforms of the quadratic (*n* = 2, *m* *=* 5, and *p* = $\frac{{15}}{{T^3}}$) and cubic (*n* = 3, *m* *=* 5, and *p* = $\frac{{20}}{{T^4}}$) polynomial FMCWs.

### Sinusoidal FMCW

When the instantaneous frequency is in a sinusoidal mode, it reaches sinusoidal FMCW. Based on Eq. (4), the phase $\varphi _0\left( t \right)$ can then be given by:

9 $$\varphi _0\left( t \right) = \frac{{2m\pi }}{T}t + \frac{{\Delta f \cdot T}}{n}\left[ {\sin \left( {\frac{{2n\pi }}{T}t} \right) + 1} \right],0 \le t \le T$$

In practical applications, the constant term in $\varphi _0\left( t \right)$ is used to ensure that the time-varying reflected phase of the STCM is always greater than 0. Then the instantaneous frequency is written as: $f_0\left( t \right) = f_c + \frac{m}{T} + \Delta f{\mathrm{cos}}\left( {\frac{{2n\pi }}{T}t} \right)$, in which $\Delta f$ is the modulation depth, and *n* and *m* are both integers. As a special case, when *m* = 0, the time-varying reflected phase of the STCM is also a sine function, and the initial frequency of FMCW is $f_c$. Figure 3c and d show the required phases $\varphi _0\left( t \right)$ and the baseband waveforms of the sinusoidal FMCW with *n* = 1, *m* = 0, $\Delta f = \frac{5}{T}$ and *n* = 2, *m* = 0, $\Delta f = \frac{5}{T}$.

Similarly, for the double sinusoidal FMCW, we have $f_0\left( t \right) = f_c + \frac{m}{T} + \Delta f_1{\mathrm{cos}}\left( {\frac{{2n\pi }}{T}t} \right) + \Delta f_2{\mathrm{cos}}\left( {\frac{{2kn\pi }}{T}t} \right)$, in which *k* is an integer, and $\Delta f_1$ and $\Delta f_2$ are the modulation depths of the two cosine functions. The corresponding phase of the STCM is defined as:

10 $$\varphi _0\left( t \right) = \frac{{2m\pi }}{T}t + \frac{{\Delta f_1 \cdot T}}{n}\left[ {\sin \left( {\frac{{2n\pi }}{T}t} \right) + 1} \right] + \frac{{\Delta f_2 \cdot T}}{{kn}}\left[ {\sin \left( {\frac{{2kn\pi }}{T}t} \right) + 1} \right],0 \le t \le T$$

Here we choose *k* = 2, *n* = 1, *m* = 0, $\Delta f_2 = 2\Delta f_1 = \frac{5}{T}$. The corresponding $\varphi _0\left( t \right)$ and the baseband waveform are shown in Fig. 3e and f.

### S-shaped FMCW

S-shaped FMCWs are also widely employed due to their advantages in pulse compression^56^. The S-shaped FMCW is a kind of FM signal whose time-frequency curve is S-shaped. It can be regarded as a weighted superposition of the linear FM signal and multiple sinusoidal FM signals with different FM rates^57,58^. Without loss of generality, we analyze the simplest case of S-shaped FMCW: the weighted superposition of a linear and a sinusoidal FM signal. The phase function $\varphi _0\left( t \right)$ of the S-shaped FMCW is given by:

11 $$\varphi _0\left( t \right) = \left( {\frac{{2m\pi }}{T} - p\pi T} \right)t + p\pi t^2 + \frac{{\Delta f \cdot T}}{n}\sin \left( {\frac{{2n\pi }}{T}t} \right),0 \le t \le T$$

and the instantaneous frequency is obtained as $f_0\left( t \right) = f_c + \left( {\frac{m}{T} - \frac{{pT}}{2}} \right) + pt + \Delta f{\mathrm{cos}}\left( {\frac{{2n\pi }}{T}t} \right)$. As an example, Fig. 3g and h exhibit the phase $\varphi _0\left( t \right)$ and the baseband waveform of the S-shaped FMCW with *n* = 1, *m* = 5, *p* = $\frac{{10}}{{T^2}}$ and $\Delta f = \frac{5}{T}$.

## Data Availability

The data that support the plots within this paper and other findings of this study are available from the corresponding author upon reasonable request.
